# DNA orientation-specific adhesion and patterning of living mammalian cells on self-assembled DNA monolayers†
†Electronic supplementary information (ESI) available: Details in experimental section and supporting figures. See DOI: 10.1039/c5sc04102c


**DOI:** 10.1039/c5sc04102c

**Published:** 2016-01-04

**Authors:** Shaopeng Wang, Xiaoqing Cai, Lihua Wang, Jiang Li, Qian Li, Xiaolei Zuo, Jiye Shi, Qing Huang, Chunhai Fan

**Affiliations:** a Division of Physical Biology & Bioimaging Center , Shanghai Synchrotron Radiation Facility , CAS Key Laboratory of Interfacial Physics and Technology , Shanghai Institute of Applied Physics , Chinese Academy of Sciences , Shanghai , 201800 , China . Email: zuoxiaolei@sinap.ac.cn; b UCB Pharma , Slough SL 1 3 WE , UK

## Abstract

To better understand cell behaviors on substrates, the precise control of density and orientation of cell-specific ligands remains a great challenge. In this study, we established an easy-to-use approach to manipulate the adhesion and patterning of mammalian cells on gold substrates. We prepared DNA self-assembled monolayers (DNA-SAMs) on gold substrates and found that the sequence-specific orientation of DNA-SAMs played an important role in modulating cell adhesion. We also found that the DNA-SAMs on gold substrates could be used as a potentially universal cell culture substrate, which showed properties similar to cationic polymers (*e.g.* poly-l lysine, PLL) substrates. Furthermore, we could manipulate cell adhesion by tuning the length of poly adenine (polyA) in the DNA sequence. We also prepared a DNA aptamer-based SAM to regulate cell adhesion by exploiting stimuli-responsive conformational change of the aptamer. By using the well-established DNA spotting technology, we patterned cells on DNA-SAMs to form a spot matrix and four English letters “CELL”. Our findings suggest that DNA-SAMs on gold substrates are potentially useful for making smart surfaces for cell studies, thus introducing a new platform for cell/tissue engineering research.

## Abstract 2

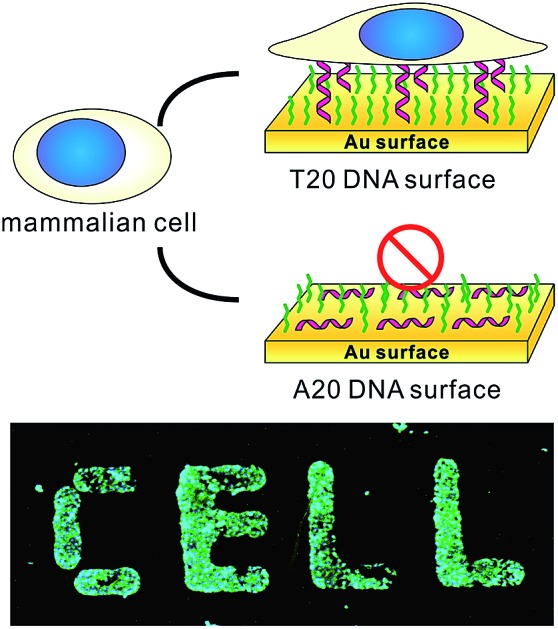
DNA orientation-specific adhesion and patterning of living mammalian cells on self-assembled DNA monolayers.

## Introduction

Controllable adhesion and patterning of living mammalian cells on substrates have attracted intense interest in many areas ranging from basic studies on cell migration and cell–cell communication to practical applications in induction of stem cells, differentiation of neurons and construction of an artificial extracellular matrix (ECM).^1^–^9^ These artificial cell substrates mimic *in vivo* extracellular environments by anchoring various chemical ligands, peptides or proteins on substrates using self-assembled monolayers (SAMs), Langmuir–Blodgett deposition, layer-by-layer assembly, or genetically engineered surface-adhesive peptides,^10^–^12^ which can repel or mediate cell adhesion to control the localization and growth of cells.^13^–^16^ Despite the widespread use of bioengineered substrates for cell/tissue engineering applications, it remains a great challenge to precisely control the density and orientation of cell-specific ligands for quantitative understanding of cell behaviors on substrates.^17^–^21^

Self-assembled monolayers (SAMs) hold great promise for controlling molecular structures on surfaces. Prior efforts in fabricating SAMs incorporating ECM-derived biomolecules (*e.g.* Arg-Gly-Asp, RGD peptide) or growth factor (*e.g.* fibroblast growth factor 2) have proven to be successful for cell adhesion with improved orderliness.^17^,^22^–^26^ Nevertheless, conventional organic molecules for making SAMs offer limited flexibility in design and synthesis for precise control of the density and orientation of the SAMs. In this regard, DNA provides a unique opportunity for making customized SAMs with high flexibility and versatility. State-of-the-art oligonucleotide synthesis technology offers high-quality DNA strands with virtually any sequence combination. More importantly, DNA-based SAMs are one of the most studied interfacial self-assembly systems, which have been extensively characterized by various techniques, including electrochemistry, fluorescence, surface-plasmon resonance, Fourier transform infrared and X-ray spectroscopy.^27^–^33^ Therefore, it is possible to precisely control the density and orientation of DNA SAMs. Previous studies have exploited SAMs with functional DNA (*e.g.* cell-specific aptamers) to capture and manipulate cells.^34^–^38^ However, little has been known on the adhesion behavior of cells on DNA SAMs that are free of cell-specific ligands. In this study, we aim to study the effect of DNA sequence-specific orientation of SAMs on the adhesion of mammalian cells. By finely tuning the base composition, density and length of DNA in the SAMs, we have established a convenient and flexible approach to manipulate the adhesion and patterning of mammalian cells on gold substrates.

## Results and discussion

**Fig. 1:**
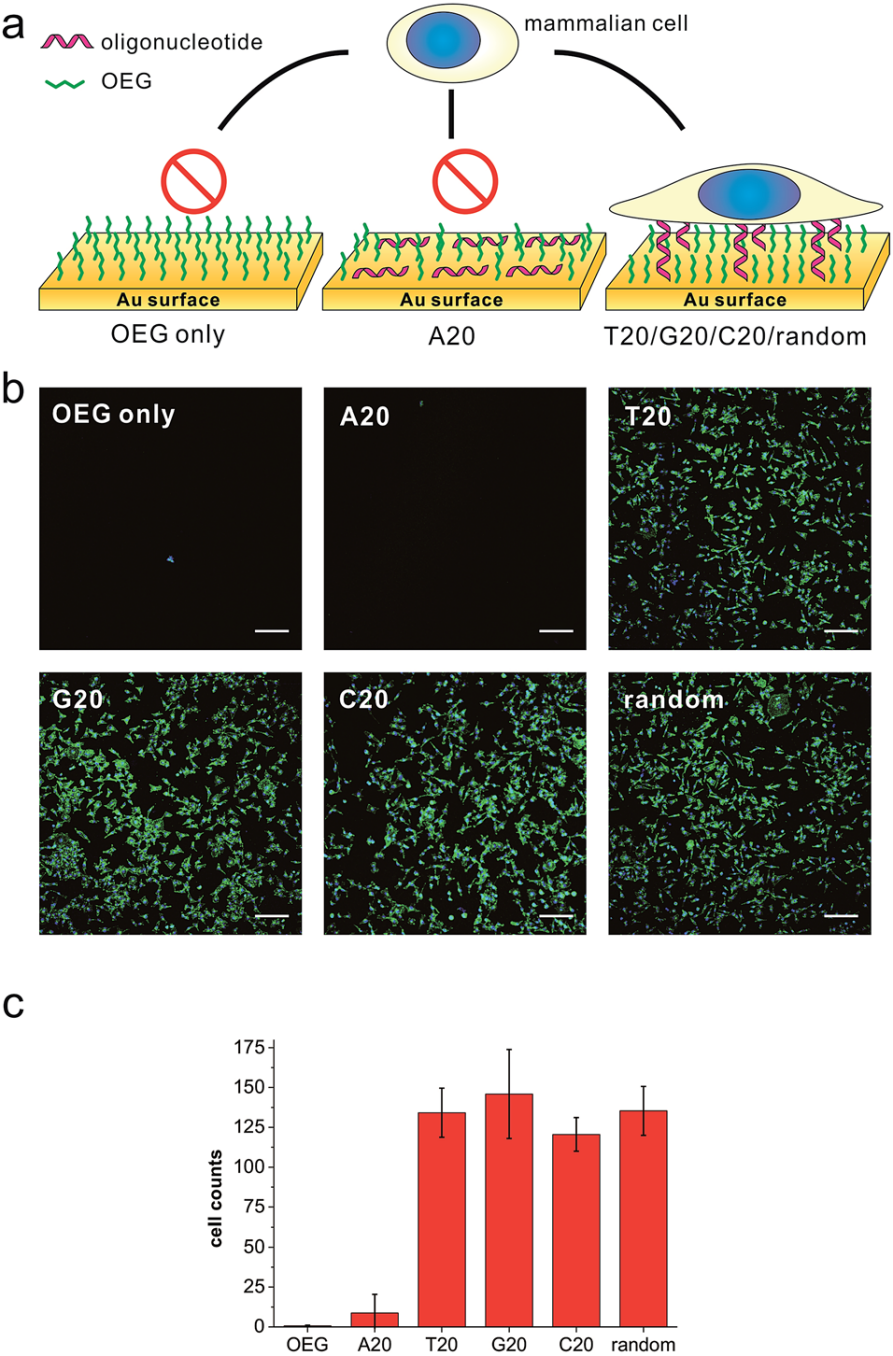
(a) The scheme of substrates derived from different DNA bases and showing different preferences to cell adhesion. (b) Fluorescence microscopy images of MCF-7 cell adhesion on different substrates. MCF-7 cell was seeded on different substrates and cultured overnight, then the cell was fixed and labeled for nuclei (blue) and actin (green) by Hoechst 33258 and phalloidin–TRITC, respectively. All the gold substrates were passivated with SH-OEG after grafting with different thiolated 20-mer DNA strands. On SH-OEG and SH-A20 substrates, no cell was observed. However, on DNA SAMs substrates formed by other thiolated DNA strands (T20, C20, G20 and a random sequence), the number of attached cells is considerable. Scale bars: 200 μm. Cells in 0.6 mm^2^ were counted. (c) The statistics of adhered cell numbers on these substrates.

We first prepared a series of DNA SAMs with different base compositions on gold substrates. Thiolated 20-mer DNA strands with five different sequences (A20, T20, C20, G20 and a random sequence) were self-assembled on gold and were then co-assembled with a hydrophilic synthetic polymer, (2-[2-(1-mercaptoundec-11-yloxy)-ethoxy]-ethanol) (SH-OEG). SH-OEG modified substrates are well known to be non-fouling, surface-resistant to the adhesion of proteins or cells. Indeed, when the SH-OEG modified substrate was incubated overnight with a human breast adenocarcinoma cell line, MCF-7, in culture medium containing 10% v/v fetal bovine serum (FBS), we did not observe any cell adhesion on this surface (Fig. 1). In contrast, when DNA SAMs were formed by mixing SH-OEG with thiolated 20-mer DNA strands (T20, C20, G20 and a random sequence), we found that cells adhered to the surface strongly, as demonstrated by fluorescent staining of actin using phalloidin–TRITC.

Cationic polymers (*e.g.* poly-l lysine, PLL) are often employed to coat substrates for cell culture in biological studies. However, DNA is an anionic polymer in nature. To explore the properties of cells grown on these reversely-charged substrates, we studied MCF-7 cells grown on T20-based SAMs and the commonly used PLL-coated substrates. Cells adhered on substrates were stained with phalloidin–TRITC (for actin) and Hoechst 33258 (for cell nuclei), and then imaged using a fluorescence microscope. As shown in Fig. S1,† cells readily adhered to the T20-based SAM in 30 min. The cell spreading behavior was similar to that on PLL substrates. We also compared the density and covered surface areas of adhered cells at different time points, which did not show any significant difference either (Fig. S1b†).

Furthermore, we investigated the expression of housekeeping genes of adhered cells to evaluate their living activities. We chose two genes that express glyceraldehyde 3-phosphate dehydrogenase (GAPDH) and β-actin proteins at constant levels in normal cells. Western blot analysis demonstrated that the expressed protein levels of GAPDH and β-actin expressed in adhered cells on DNA-SAM and PLL substrates were nearly the same (Fig. S2†), which provides evidence for the living state of cells at the molecular level. Our further studies on four commonly used cell lines (PC12, HaCaT, HeLa, and CHO) revealed similar behavior in morphology, density and surface areas of adhered cells on both DNA-SAMs and PLL (Fig. S3 and S4†). Therefore, despite the reversed charge state of DNA-SAMs, they are suitable for cell adhesion and growth and can be used as a potentially universal cell culture substrate.

**Fig. 2:**
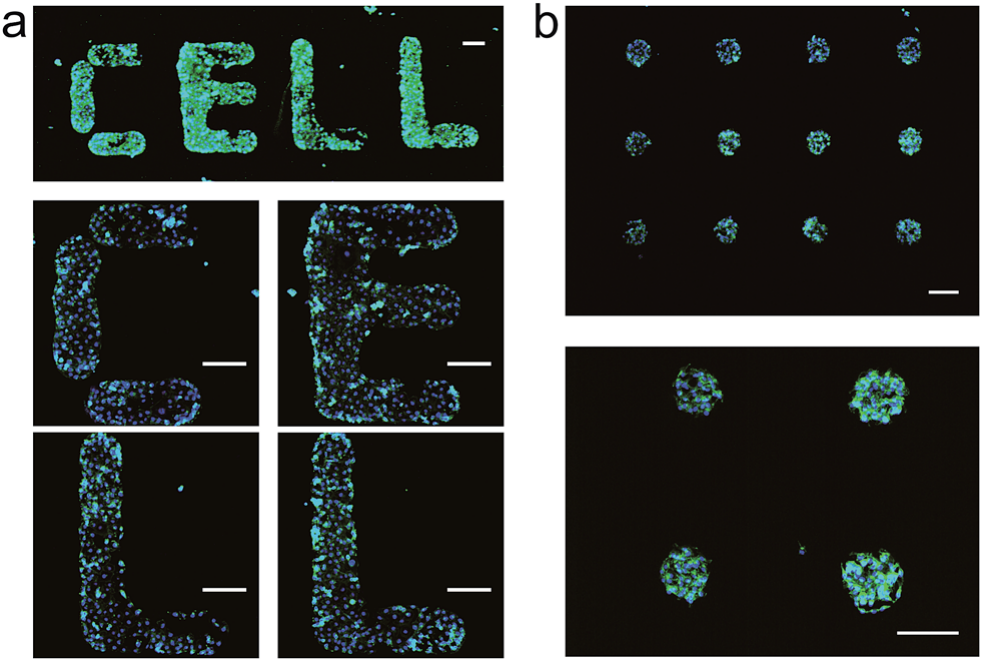
Patterns of living cells on DNA-SAM substrate. SH-T20 was spotted on gold substrate by a microarrayer to form a spot matrix (500 μm distance) or four English letters: “CELL”; after passivation with SH-OEG; MCF-7 cells were seeded and cultured overnight. After staining with Hoechst 33258 and phalloidin–TRITC, cell patterns were observed by microscope. (a) English letters “CELL” built by patterned cells. (b) Cell spot matrix. Scale bars: 200 μm.

Having established that DNA-SAMs provide a new type of substrate for cell adhesion, we attempted to pattern cells on substrates by exploiting the convenience in spotting DNA arrays of arbitrary shapes. We spotted T20 on a gold substrate to form a spot matrix. When MCF-7 cells were seeded on this substrate, we observed that cells specifically adhered to the spotting areas with T20, forming an array of cells exhibiting green fluorescence of phalloidin–TRITC that is stained on actin (Fig. 2b). As a further demonstration of the cell patterning power of this DNA-based method, we spot four English letters, “CELL”, using T20 and again seeded cells on the substrate. Then, we found these letters (CELL) emitted green fluorescence of phalloidin–TRITC under the fluorescence microscope (Fig. 2a). Staining of the nuclei with Hoechst 33258 provides additional evidence for the formation of the cell pattern. In addition, we found that cells distributed relatively evenly in these spotting areas. Therefore, we can precisely control the spatial arrangement of living cells on substrates to form predefined patterns using DNA-SAMs. This ready-to-use method should be useful for cell patterning in cell/tissue engineering, cell-based sensing and drug discovery.^4^,39a

Furthermore, we started to investigate DNA SAMs assembled with thiolated A20. Previous studies have established that consecutive adenines possess greater affinity on gold as compared to other base combinations.^28^ As a result, polyA adopts a “lying-flat” orientation on gold, which is distinctly different from other DNA sequences that take the upright orientation. Interestingly, when we prepared A20-based DNA SAMs, the surface repelled cell adhesion as it did with pure SH-OEG (Fig. 1). Such remarkable difference in cell adhesion inspired us to investigate the effects of DNA sequences on cell adhesion. To investigate the difference in orientation of polynucleotides in DNA SAMs, we employed Cy3-tagged T20 and A20 to co-assemble with OEG on gold substrates. Because gold is a well known high efficiency quencher for fluorescence, fluorescence imaging can provide a sensitive measurement of the orientation of DNA based on the distance-dependent gold-quenched fluorescence. We observed intense fluorescence on the T20-based SAM under the fluorescence microscope, whereas no fluorescence was found on the A20-based or pure OEG SAMs (Fig. S5†). This striking difference suggests that A20 and T20 take distinctly different orientations on gold. We reason that the presence of SH-OEG disrupts weak interactions between T20 and gold, which leaves the tagged Cy3 far away from the surface. In contrast, the strong interactions between A20 and gold are little perturbed by SH-OEG, which maintains the flat orientation of A20.^28^,^29^ Consequently, Cy3 is kept close to gold and efficiently quenched.

**Fig. 3:**
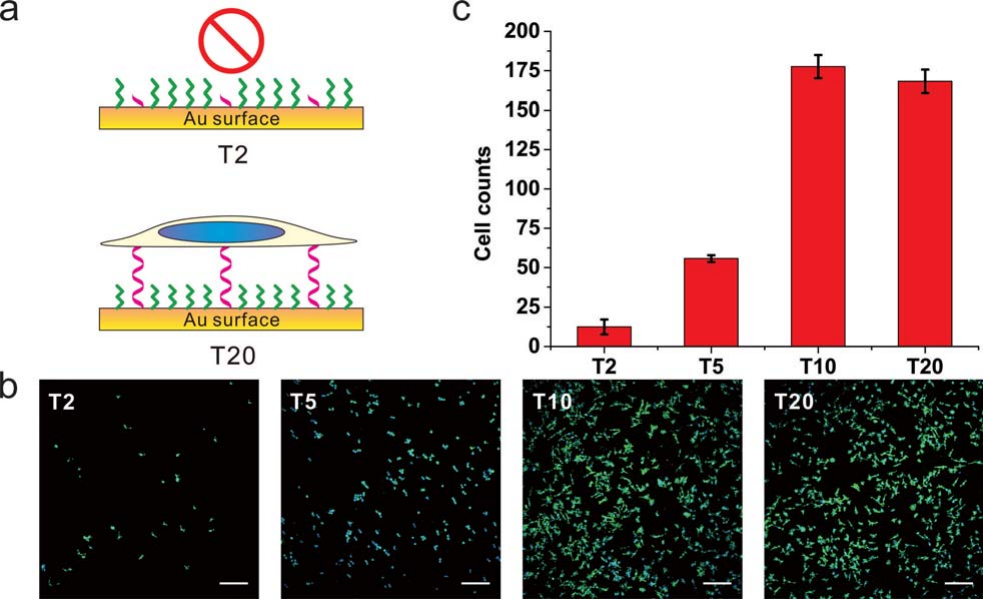
The (a) scheme, (b) fluorescence microscopy images and (c) statistics of adhered cell numbers of MCF-7 cells on gold surface grafting with thiol-oligoT of different lengths. DNA SAMs were formed by thiol-oligoT with different lengths (T20, T10, T5, T2) followed by passivation with SH-OEG, MCF-7 cell was cultured on those SAMs overnight and stained with Hoechst 33258 and phalloidin–TRITC. Cell number was analyzed by ImageJ. Compared to T20 SAMs, no significant difference on cell density is observed for T10 SAMs. However, cell number decreased significantly for T5 SAMs and almost no cells attached for T2 SAMs. Cells in 0.6 mm^2^ were counted. Scale bars: 200 μm.

To further substantiate the observation that the orientation of DNA SAMs exerts a great influence on cell adhesion, we varied the length of non-adsorbed polyT to co-assemble with SH-OEG. Our rationale is that short polyT would be embedded in the OEG layer, resembling surface-adsorbed polyA, whereas long polyT would protrude out of the OEG layer, allowing efficient cell adhesion. A series of polyT of different lengths (T2, T5, T10 and T20) of the same concentration was co-assembled with SH-OEG on gold. As shown in Fig. 3, cell adhesion on the T10-based SAM was similar to that on T20. However, the density of adhered cells decreased remarkably on the T5-based SAM, and negligible cells were observed on the T2-based SAM. This length-dependent trend of cell adhesion supports our proposed rationale. When the height of polyT was comparable with or longer than the height of OEG (2.4 nm), the SAM adopts the cell adhesion property of DNA. On the contrary, T2 and T5 are shorter than OEG and embedded in the OEG layer, which results in SAMs that are resistant to the adhesion of cells. Therefore, although polyT and polyA have distinctly different properties, our studies indicate that the orientation, instead of the sequence, dominate their cell adhesion ability.

Furthermore, we studied the effect of DNA density on cell adhesion. T20 with different concentrations (2 μM, 1 μM, 500 nM and 100 nM) was co-assembled with SH-OEG on gold. When the assembly concentration of T20 was higher than 100 nM, we observed significant adhesion of MCF-7 cells with comparable amount to each other (Fig. S6†). Remarkably, very few cells were adhered to the T20-based SAM when the assembly concentration of T20 was decreased to 100 nM. We reason that this nearly “all-or-none” effect should also come from the orientation effect. Given that the contour length of T20 is ∼7 nm, whereas the threshold DNA density meant ∼5 nm interspace among those DNA strands. When the contour length of T20 is longer than the inter-strand distance, T20 tends to stay upright due to strong lateral electrostatic repulsion; when the contour length of T20 is shorter than the inter-strand distance, the lack of lateral interactions makes DNA strands lie down on the OEG layer, which is an orientation unfavorable for cell adhesion.

Our extensive studies on the effects of DNA density, length and sequence on the adhesion properties of cells inspired us to manipulate the cell adhesion by programmably tuning the length of poly adenine (polyA) in the DNA sequence, through which we expected to tune the cell adhesion on DNA SAMs. We inserted different lengths of polyA (from 0 to 80 nt) between thiol group and random sequence, which were used to prepare DNA SAMs (Fig. S7 and S8†). When the polyA length was shorter than 40 nt, we observed significant adhesion of MCF-7 cells with comparable amount to that of the random sequence. Remarkably, very few cells were adhered to the DNA SAM when the polyA length was longer than 40 nt.

**Fig. 4:**
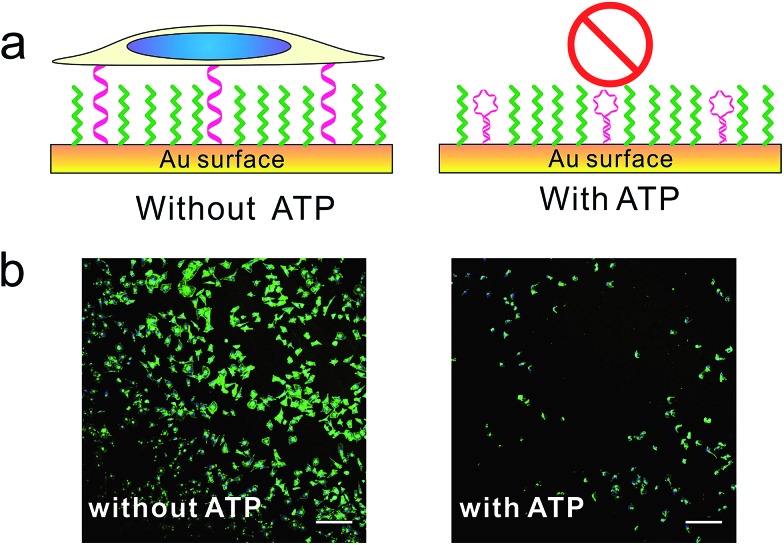
The (a) scheme and (b) fluorescence microscopy images of control cell adhesion through ATP. MCF-7 cell was seeded on DNA SAMs either in the presence or absence of ATP overnight. Cells were fixed and nuclei (blue) and actin (green) were labelled with Hoechst 33258 and phalloidin–TRITC. Scale bars: 200 μm. We constructed this ATP-responsive DNA SAM by grafting ATP's aptamer on gold substrate. Without ATP, the aptamer adopted unfolded state and tended to stay upright due to strong lateral electrostatic repulsion, which has high cell adhesion property. With ATP, the conformational change of aptamer forced it to adopt a folded structure, which was unfavorable for cell adhesion.

Furthermore, we designed a stimuli-responsive DNA SAM by constructing an ATP aptamer-based39*b* DNA SAM. In the absence of ATP, the aptamer adopted an unfolded, extended state and tended to stay upright due to strong lateral electrostatic repulsion, which showed a high cell adhesion property. In the presence of ATP, the conformational change of aptamer forced it to fold into a tertiary structure, which was unfavorable for cell adhesion. As a result, we observed a remarkably reduced level of cell adhesion (Fig. 4, S9†).

**Fig. 5:**
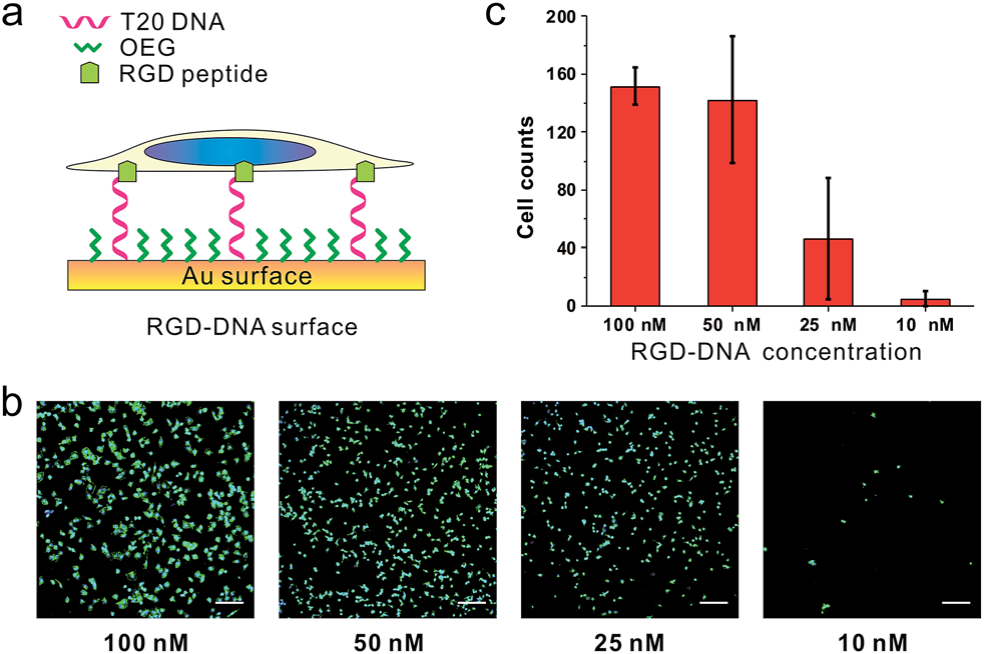
The (a) scheme, (b) fluorescence microscopy images and (c) statistics of cells from RGD-DNA surfaces of different DNA densities. RGD was coupled with DNA through SMCC. The RGD-DNA was annealed with thiol-labeled complementary DNA, the product of which was then used to build SAMs on the gold substrate with different concentrations (100 nM, 50 nM, 25 nM, and 10 nM). Cell nuclei (blue) and actin (green) of MCF-7 cell adhesion on different SAMs were labeled. Cells in 0.6 mm^2^ were counted. Scale bars: 200 μm.

Because we can finely modulate cell adhesion by changing the density, length and sequence of DNA, we further explore whether we can use these DNA-SAMs to control cell adhesion molecules (CAMs) that are *trans*-membrane proteins located on the cell surface. To this end, we modified SH-DNA with RGD that can bind specifically to integrin, a well-known CAM that bridges cell–cell and cell–extracellular matrix (ECM) interactions.^28^ We prepared DNA SAMs using RGD coupled DNA with a series of concentrations. When the assembly concentration was 100 nM, we observed significant adhesion of cells, which is in direct contrast with that on T20-based SAMs free of RGD (Fig. 5). This suggests that the strong interactions between RGD and integrin on the cell membrane force DNA strands to take an upright orientation,^37^ even in the absence of inter-probe lateral interactions at low densities. We further observed concentration-dependent cell adhesion when the RGD is present, which is also different from the “all-or-none” behavior observed in RGD-free SAMs.

## Conclusions

In this study, we have demonstrated that DNA-SAMs can be used as a type of facile and highly tunable substrate for cell culture. Furthermore, we can manipulate cell adhesion by tuning the length of poly adenine (polyA) in the DNA sequence and, in turn, the cell adhesion on the DNA SAM can be programmably regulated. We also prepared a DNA aptamer-based SAM to regulate cell adhesion by exploiting stimuli-responsive conformational change of the aptamer. These DNA-SAM-based substrates showed comparable properties for cell adhesion and growth to commercially available PLL substrates. Our extensive studies on the effects of DNA density, length and sequence on the adhesion properties of cells reveal that DNA orientation on gold plays an important role in modulating cell adhesion. This provides a versatile approach to manipulate the adhesion of cells on substrates, which is potentially useful for making smart surfaces for cell studies. We also demonstrated the ability to precisely control the density of RGD, which should be readily adaptable in cell/tissue engineering. By taking advantage of the well-established DNA spotting technology, we can readily form microarrays of living cells with predefined patterns, which is useful for manipulating the growth and differentiation of cells and realizing artificial extracellular matrix and even tissues/organs on chips.^16^,^39^–^42^

## Experimental

### Materials and cell culture

DNA oligonucleotides were purchased from Takara (purified by HPLC). OEG 2-[2-(1-mercaptoundec-11-yloxy)-ethoxy]-ethanol (HS-C_11_-EG_2_) was purchased from Prochimia. SH-PEG (MW 5000) was purchased from Laysan Bio. Inc. Mercaptohexanol (MCH), Hoechst 33258, phalloidin–tetramethylrhodamine B isothiocyanate (TRITC) and SMCC were purchased from Sigma. Cyclic peptide RGDfK-NH_2_ was purchased from Peptides International, Inc.

DNA sequences are as follows:

SH-A20: SH-AAAAAAAAAAAAAAAAAAAA

SH-T20: SH-TTTTTTTTTTTTTTTTTTTT

SH-G20: SH-GGGGGGGGGGGGGGGGGGGG

SH-C20: SH-CCCCCCCCCCCCCCCCCCCC

SH-random sequence: SH-GTGTCGTGCCTCCGTGCTGTG

SH-DNA: SH-CACAGCACGGAGGCACGACAC

SH-A10-random: SH-AAAAAAAAAAGTGTCGTGCCTCCGTGCTGTG

SH-A20-random: SH-AAAAAAAAAAAAAAAAAAAAGTGTCGTGCCTCCGTGCTGTG

SH-A30-random: SH-AAAAAAAAAAAAAAAAAAAAAAAAAAAAAAGTGTCGTGCCTCCGTGCTGTG

SH-A40-random: SH-AAAAAAAAAAAAAAAAAAAAAAAAAAAAAAAAAAAAAAAAGTGTCGTGCCTCCGTGCTGTG

SH-A80-random: SH-AAAAAAAAAAAAAAAAAAAAAAAAAAAAAAAAAAAAAAAAAAAAAAAAAAAAAAAAAAAAAAAAAAAAAAAAAAAAAAAAGTGTCGTGCCTCCGTGCTGTG

SH-ATP aptamer: SH-ACCTGGGGGAGTATTGCGGAGGAAGGT

TM buffer was prepared from 10 mM of Tris and 5 mM of MgCl_2_ (pH = 8.0). All solutions were prepared with deionized water.

Cells were cultured in medium supplemented with 10% fetal bovine serum, penicillin/streptomycin (100 units per mL) and l-glutamine (2 mM) at 37 °C in humidified environment containing 5% CO_2_. For different cells, different media were used. MCF-7 was cultured with RPIM 1640; HeLa, PC12 and HacaT were cultured with DMEM; CHO was cultured with F12-K.

### Preparation of DNA-SAMs (self-assembled monolayers)

Gold films were cleaned by sonicating in ethanol and water for 15 min, respectively, followed by irradiation under UV for 15 min to make them asepsis. 3 μM of thiol-modified DNA in TM buffer was added on the gold surface for 4 h for immobilization. After removing surplus liquid, the gold surface was immersed in 4 mM of OEG for 4 h, so that the gold surface that was not covered by DNA was grafted with a monolayer of OEG. For cell experiments, the gold films were washed extensively with PBS.

For cell pattern experiments, a DNA microarrayer was used to spot SH-T20 on gold substrate with a distance of 500 μm for four English letters: “CELL”. After assembly for 4 h, gold substrates were passivated with OEG.

For RGD-DNA-SAMs, RGDfK-NH_2_ and SH-DNA were first coupled through SMCC (a hetero-bifunctional crosslinker). After annealing with 5′-thiol-labeled complementary DNA strands (SH-random sequence) in TM buffer, the product was added on the gold surface to assemble.

In the experiments of controlled cell adhesion by ATP, 1 μM thiol-labeled DNA was incubated at 37 °C with or without the presence of 1 mM ATP for half an hour. Then DNA was grafted on the gold surface for an hour followed by passivation with SH-PEG5000 for 4 hours to perform cell experiments.

### Characterization of fluorescence-labeled DNA on gold surface by microscopy

DNA was assembled on the gold surface as described above. Briefly, 3 μM of Cy3-labeled thiol-modified DNA in TM buffer was added on the surface for 4 h for immobilization. After passivation in 4 mM OEG for 4 h, the surface was imaged with fluorescence microscopy (Leica epi-fluorescence with EMCCD).

### Cell experiments

Cells were seeded on PLL-coated coverslips or DNA self-assembled monolayers in 24-well culture plates at a density of 6 × 10^4^ cells per well for HeLa, MCF-7 and 10 × 10^4^ cells per well for PC12, HacaT and CHO. For the ATP aptamer experiment, MCF-7 cells were seeded either with or without 1 mM ATP. At specific time points, cells were washed three times with pre-warmed PBS, and fixed with paraformaldehyde/sucrose (4% w/v) in PBS at room temperature for 15 min. Then cells were washed with PBS and permeated with TritonX-100 (0.1%) in PBS for 15 min. After blocking with BSA (1%) for 30 min, the actin cytoskeleton was labeled with 1 μg mL^–1^ of phalloidin–TRITC for 20 min and cell nuclei were labeled with Hoechst 33258 for 10 min at room temperature. Finally, the cells were washed extensively with PBS before observation by microscopy.

To study the process of cell adhesion on the DNA surface, cells were fixed at various time points (0.5 h, 1 h, 2 h, and 4 h) after seeding.

### Western blotting

MCF-7 cells were seeded on DNA-SAMs and PLL-coated substrates and cultured overnight. Cells were then harvested and cell numbers counted. For protein extraction, equal numbers of cells were used. Whole lysate was resolved by SDS-PAGE, transferred onto PVDF membranes. GADPH and actin were immunoblotted by specific primary antibodies and secondary antibodies (Santa Cruz).

### Statistical analysis

Cell numbers were determined by counting cell nuclei in the 200× magnified field of view and projected cell areas were determined based on an algorithm using ImageJ. Data are presented as mean ± SD.

## Supplementary Material

Supplementary information
