# Supplementary material for: DNA orientation-specific adhesion and patterning of living mammalian cells on self-assembled DNA monolayers
Source: Chem Sci. 2016 Jan 4;7(4):2722–7. doi: 10.1039/c5sc04102c (PMC5477012; doi:10.1039/c5sc04102c)
Supplement: Supplementary file 1 [file SC-007-C5SC04102C-s001.pdf]

Electronic Supplementary Material (ESI) for Chemical Science.
This journal is © The Royal Society of Chemistry 2016

DNA orientation-specific adhesion and patterning of living mammalian cells on self-assembled DNA monolayers

Shaopeng Wang,[a,+] Xiaoqing Cai,[a,+] Lihua Wang,[a] Jiang Li,[a] Qian Li,[a] Xiaolei Zuo,*[a] Jiye Shi,[a, b] Qing Huang,[a] and Chunhai Fan[a]

1. Supporting figures

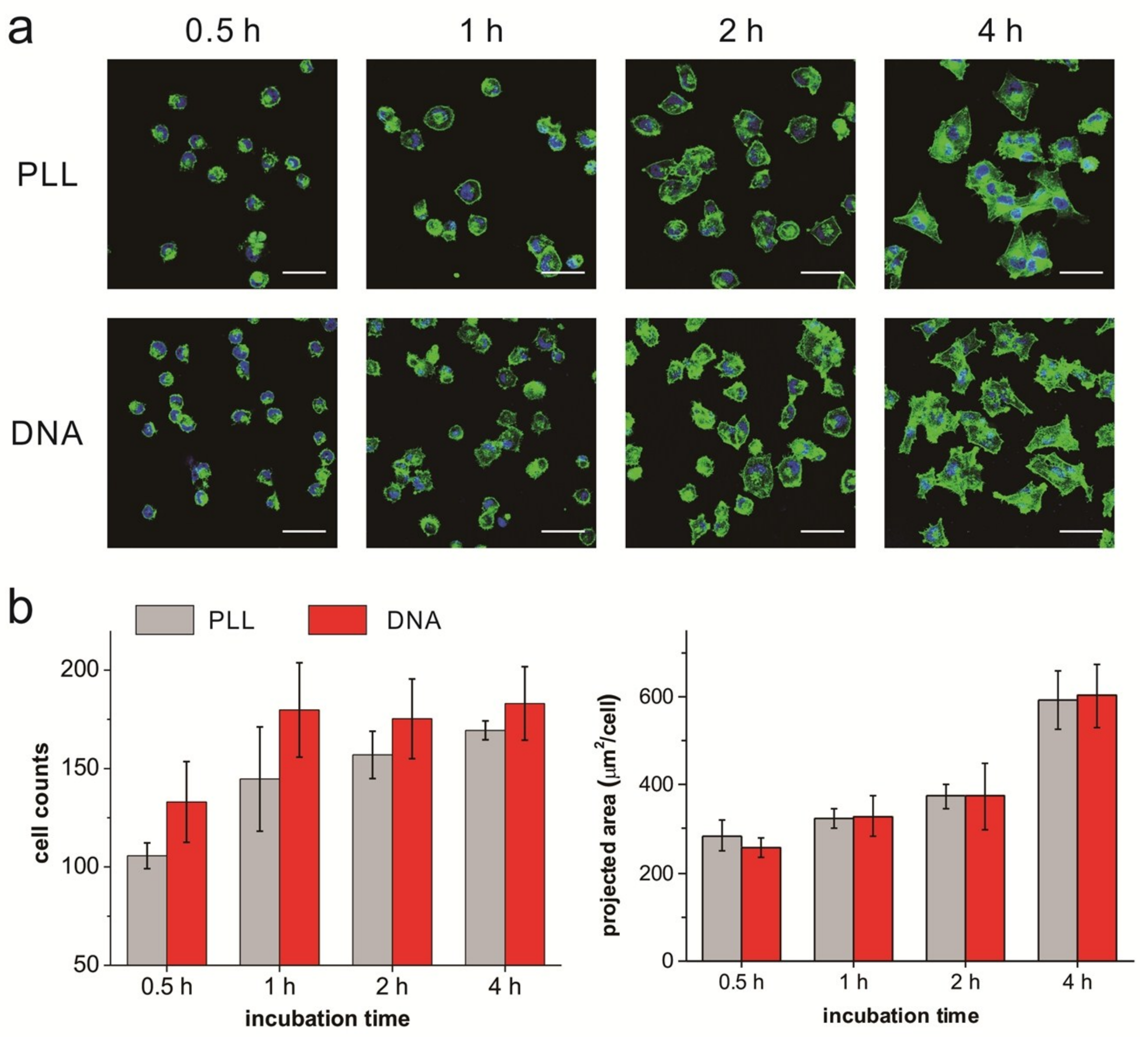

Figure S1. Microscopic images (a) and statistic cell number/cell area (b) of MCF-7 cells at different time points on DNA surface and PLL-coated substrate. MCF-7 cell was seeded either on DNA SAMs formed by SH-T20 or PLL-coated substrate, at different time point (0.5h, 1h, 2h, 4h) cell was fixed, cell number and cell area were calculated by nuclei (blue) and actin (green) labeling with Hochest 33258 and phallodin-TRITC based on a software (imaging J). The process of cell adhesion on DNA surface is similar to that on PLL-coated substrate, and no significant difference was found either on cell number or projected cell area between PLL-coated substrate and DNA surface. Cells in 0.6 mm^2 were counted. Scale bars: 50μm.

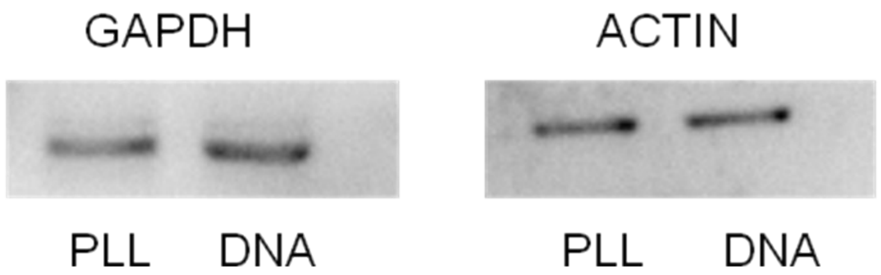

Figure S2. Western blot analysis of the expression of two housekeeping genes (GAPDH and actin) of MCF-7 cells cultured on DNA-SAM surface and PLL-coated substrates. MCF-7 cells cultured on PLL-coated substrates and DNA-SAMs overnight were harvested. Equal numbers of cells were used for protein extraction.

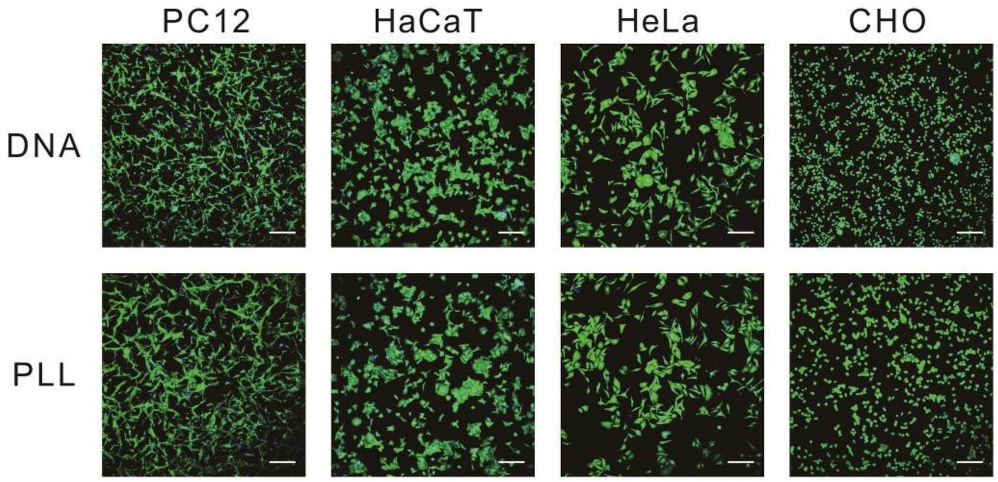

Figure S3. Microscopic imaging of different cell lines on DNA-SAM surface and PLL-coated substrates. Four types of cells were seeded on DNA-SAMs or PLL-coated substrates and cultured overnight. Cell nuclei (blue) and actin (green) were labelled with Hochest 33258 and phallodin-TRITC, respectively. Scale bars: 200 μm.

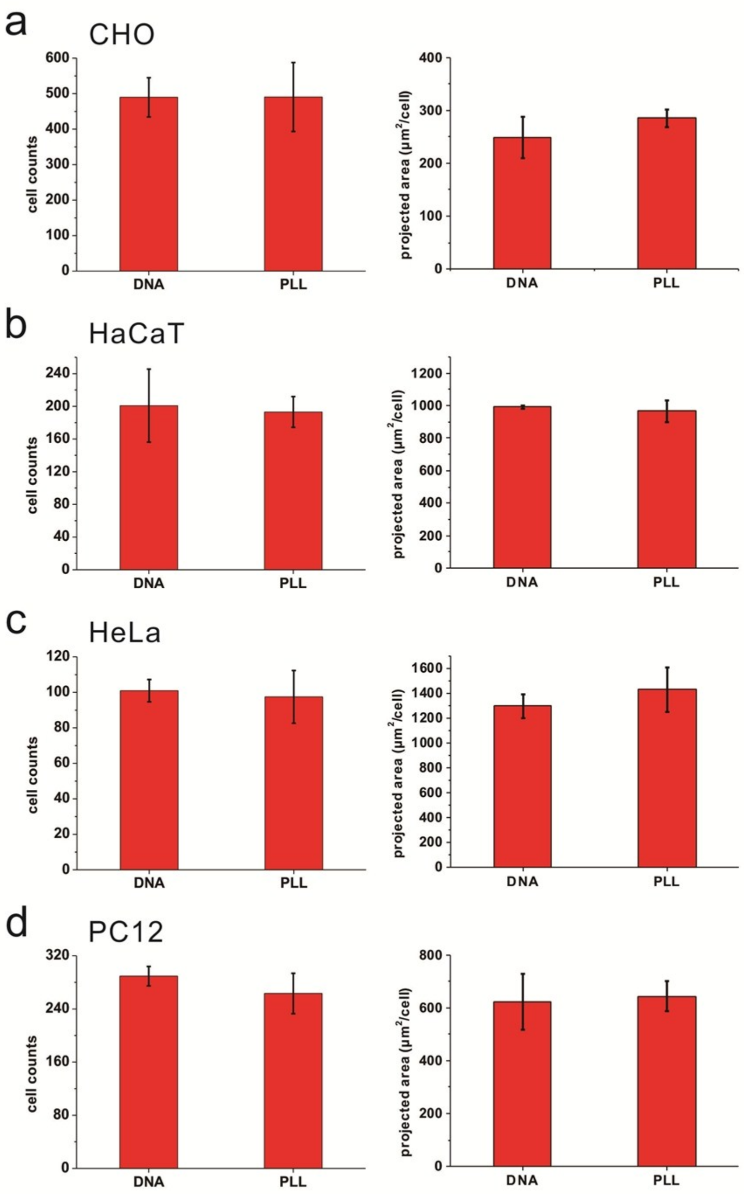

Figure S4. Statistic analysis of cell numbers and projected cell areas of (a) CHO, (b) HaCaT, (c) HeLa and (d) PC12 cells cultured on DNA-SAM surface and PLL-coated substrates. Cells in 0.6 mm^2 were counted. Projected cell areas were calculated using actin imaging by Image J.

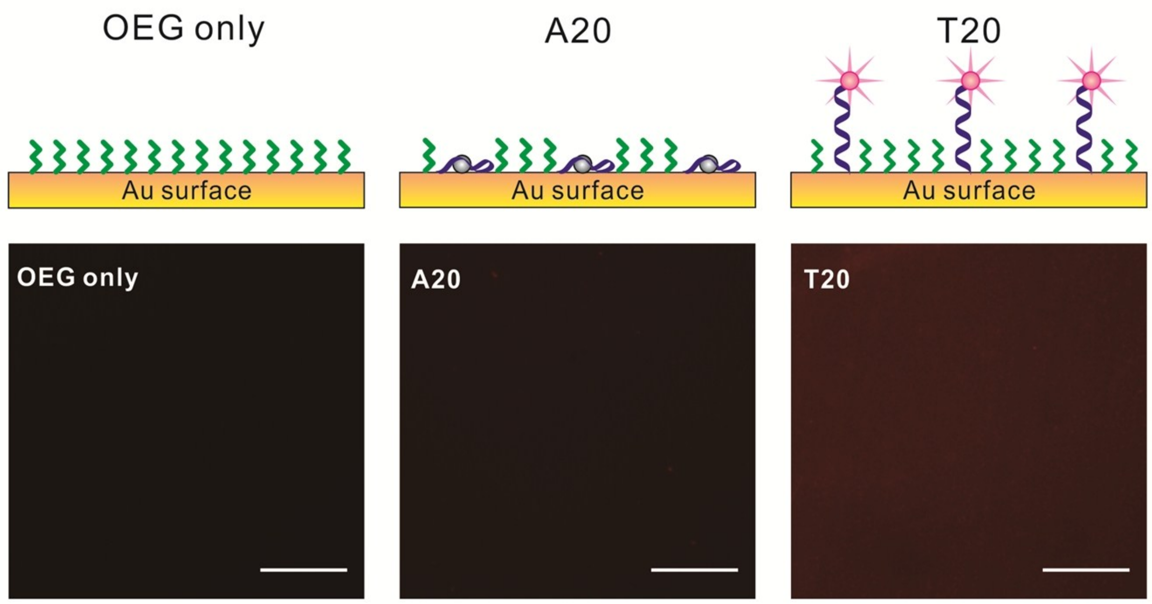

Figure S5. The scheme (top) and the fluorescence (bottom) of gold surfaces modified with OEG only, A20 and T20, respectively. A20 and T20 labeled at 5' and 3' end with thiol and Cy3 were grafted on gold surface. After the passivation with SH-OEG, the fluorescence intensity was compared with fluorescence microscopy. Scale bars: 20 μm.

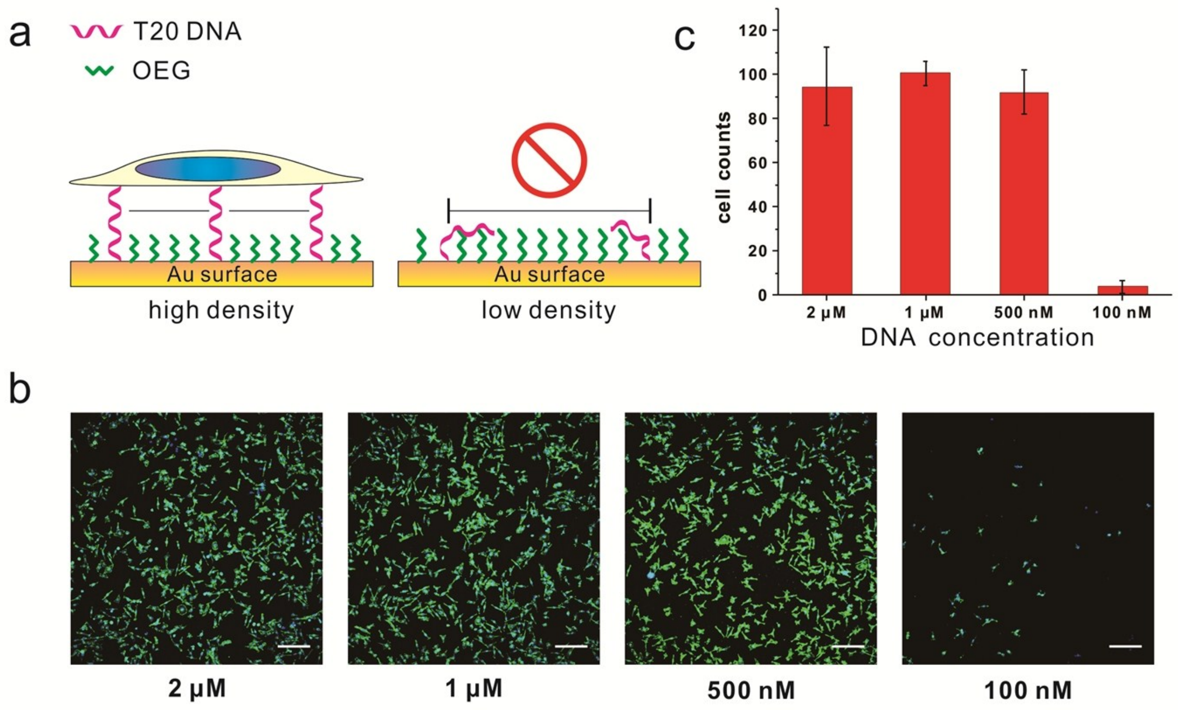

Figure S6. The scheme (a) and fluorescence microscopic images (b) of cells adhesion on DNA SAMs with different DNA density. DNA density on gold substrate was controlled by grafting thiol-oligoT with different concentration (2μM, 1μM, 500nM, 100nM). MCF-7 cell was seeded on different substrates overnight before fixed and nuclei (blue) and actin (green) were labeled with Hochest 33258 and phallodin-TRITC. (c) Statistic cell number of MCF-7 cells adhesion on DNA-SAM surface with different DNA grafting densities. Cells in 0.6 mm^2 were counted. Scale bars: 200 μm.

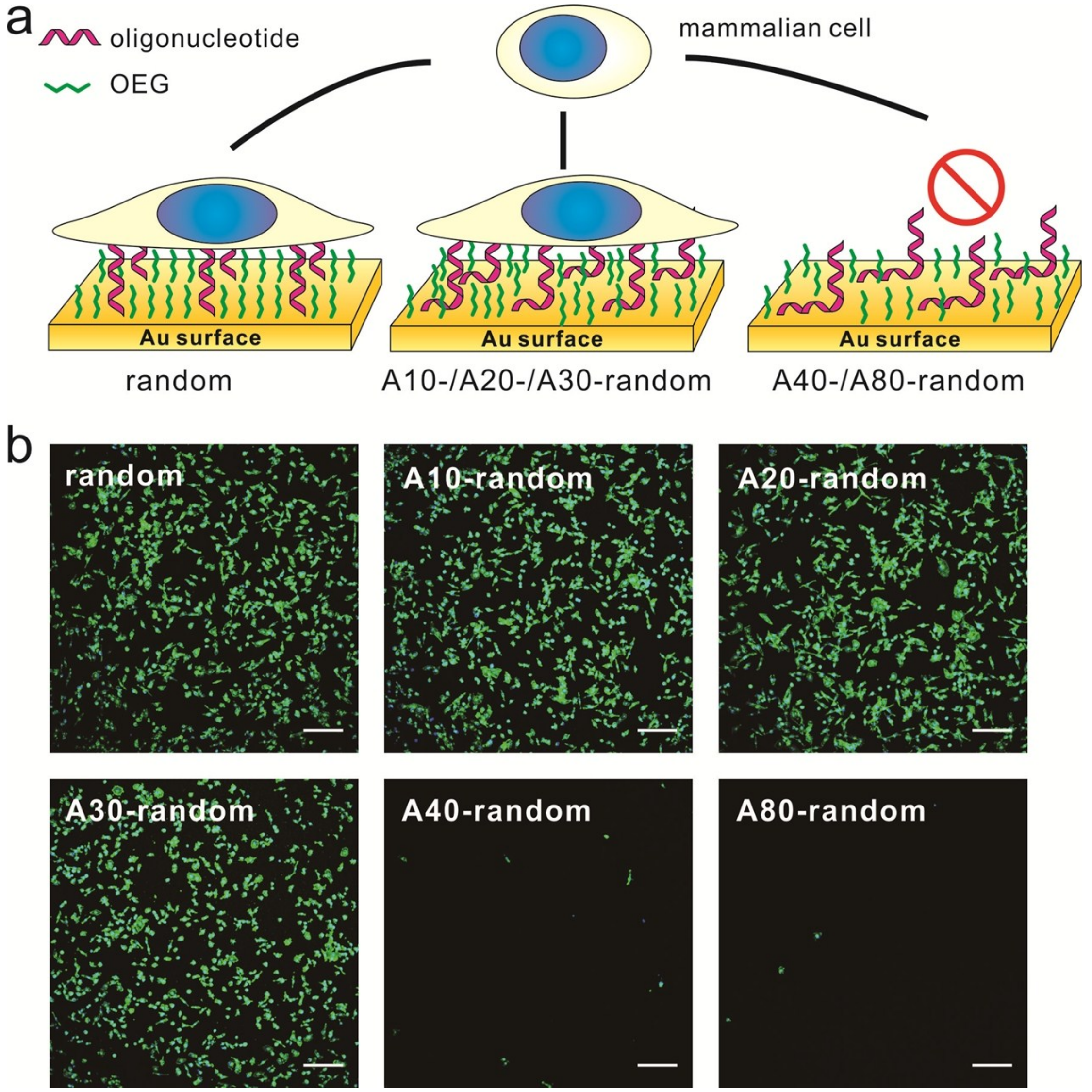

Figure S7. The scheme (a) and fluorescence microscopic images (b) of cells adhesion on DNA SAMs with different DNA density by tuning the polyA length in DNA sequence. DNA density on gold substrate was regulated by grafting thiolated DNA contain different length of polyA sequence (0nt, 10nt， 20nt, 30nt, 40nt, 80nt) between thiol group and random sequence. MCF-7 cell was seeded on different substrates overnight before fixed and nuclei (blue) and actin (green) were labeled with Hochest 33258 and phallodin-TRITC. Scale bars: 200 µm. When the polyA length was shorter than 40 nt, we observed significant adhesion of MCF-7 cells with comparable amount. Remarkable, very few cells were adhered to the DNA SAM when the polyA length was longer than 40 nt.

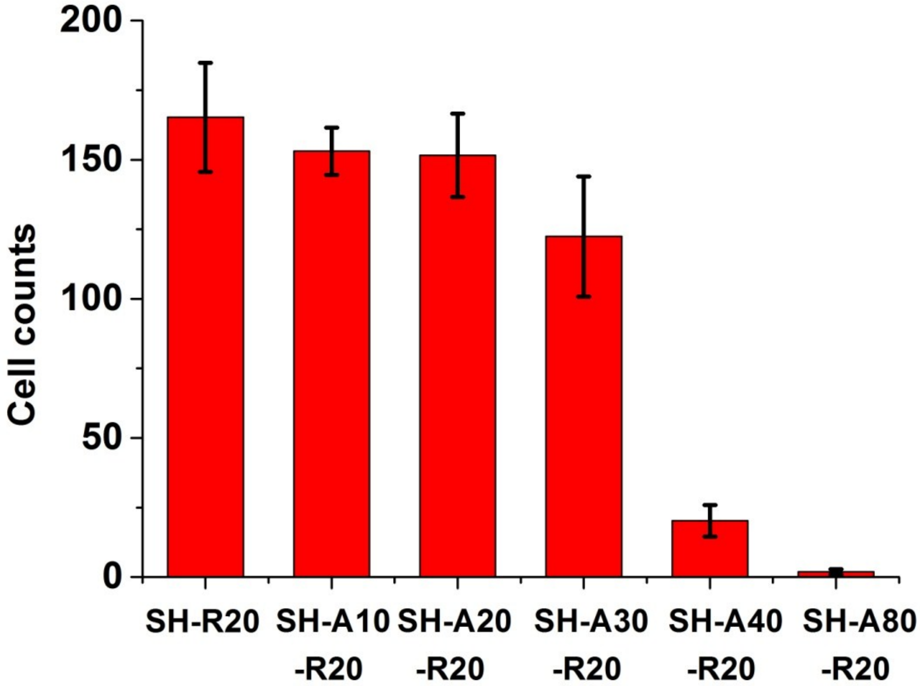

Figure S8. Statistic of cell numbers adhesion on thiolated DNA sequence with different length of poly A.

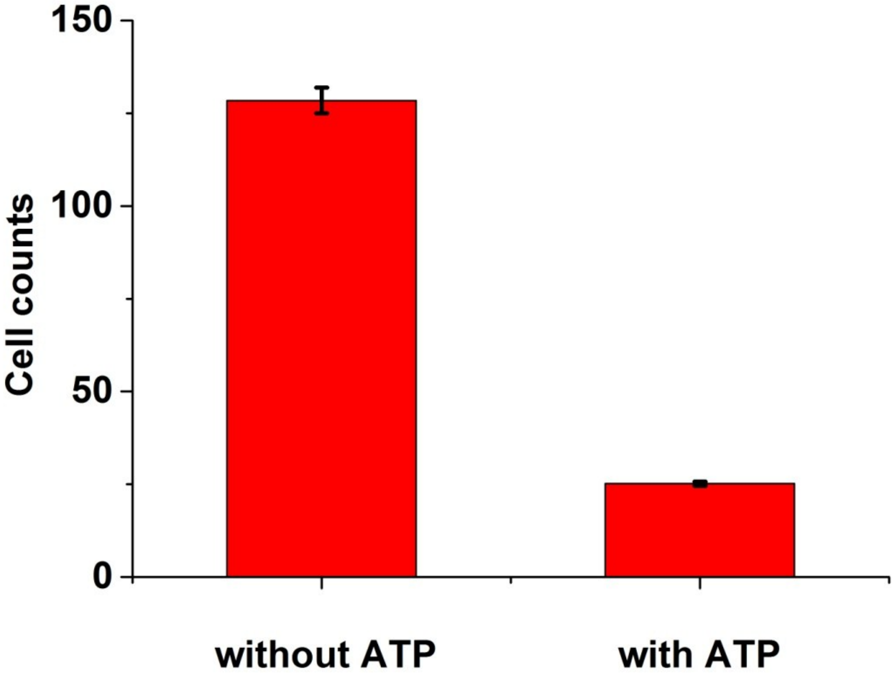

Figure S9. Statistic of cell numbers adhesion on DNA SAMs with or without ATP.

2. Materials and methods

Materials and cell culture

DNA oligonucleotides were purchased from Takara (purified by HPLC). OEG 2-[2-(1-mercaptoundec-11-yloxy)-ethoxy]-ethanol ($HS-C_{11}-EG_2$) was purchased from Prochimia. SH-PEG (MW 5000) was purchased from Laysan Bio. Inc. Mercaptohexanol (MCH), Hoechst 33258, Phalloidin-Tetramethylrhodamine B isothiocyanate and SMCC were purchased from Sigma. Cyclic peptide RGDfK-NH_2 was purchased from Peptides International, Inc.

DNA sequences are as following:

SH-A20: SH-AAAAAAAAAAAAAAAAAAAA
SH-T20: SH-TTTTTTTTTTTTTTTTTTTT
SH-G20: SH-GGGGGGGGGGGGGGGGGGGG
SH-C20: SH-CCCCCCCCCCCCCCCCCCCC
SH-random sequence: SH-GTGTCGTGCCTCCGTGCTGTG
SH-DNA: SH-CACAGCACGGAGGCACGACAC
SH-A10-random: SH-AAAAAAAAAAGTGTCGTGCCTCCGTGCTGTG
SH-A20-random: SH-AAAAAAAAAAAAAAAAAAAAGTGTCGTGCCTCCGTGCTGTG
SH-A30-random: SH-
AAAAAAAAAAAAAAAAAAAAAAAAAAAAAAGTGTCGTGCCTCCGTGCTGTG
SH-A40-random: SH-
AAAAAAAAAAAAAAAAAAAAAAAAAAAAAAAAAAAAAAAAGTGTCGTGCCTCC
GTGCTGTG
SH-A80-random: SH-
AAAAAAAAAAAAAAAAAAAAAAAAAAAAAAAAAAAAAAAAAAAAAAAAAAAAAA
AAAAAAAAAAAAAAAAAAAAAAAAAAGTGTCGTGCCTCCGTGCTGTG
SH-ATP aptamer: SH- ACCTGGGGGAGTATTGCGGAGGAAGGT

TM buffer was prepared from 10 mM of Tris and 5 mM of $MgCl_2$ (pH=8.0). All solutions were prepared with deionized water.

Cells were cultured in medium supplemented with 10% fetal bovine serum, penicillin/streptomycin (100 units/mL) and L-glutamine (2 mM) at 37 ^{o}C in humidified environment containing 5% CO_2. For different cells, different medium were used. MCF-7 was cultured with RPIM 1640; Hela, PC12 and HacaT were cultured with DMEM; CHO was cultured with F12-K.

Preparation of DNA-SAMs (self-assembled monolayers)

Gold films were cleaned by sonicating in ethanol and water for 15 min, respectively, followed by irradiating under UV for 15 min to make them asepsis. 3 μM of thiol-modified DNA in TM buffer was added on the gold surface for 4 h for immobilization. After removing surplus liquid, the gold surface was immersed in 4 mM of OEG for 4 h, so that the gold surface that was not covered by DNA was grafted with a monolayer of OEG. For cell experiment, the gold films were washed extensively with PBS.

For cell pattern experiment, DNA microarrayer was used to spot SH-T20 on gold substrate with a distance of 500 μm. After assembly for 4 h, gold substrates were passivated with OEG.

For four English letters: “CELL”, we used the DNA microarrayer which can be programed to spot arbitrary shapes. We adjusted the distance between two spots to the minimum distance (100nm), therefore, when DNA solution were spotted on the gold surface, the spots connected with each other to form the continuous letters. We designed the same height and width for the four letters to ensure that they can be spotted on the same row.

For RGD-DNA-SAMs, RGDfK-NH_2 and SH-DNA were first coupled through SMCC (a hetero-bifunctional crosslinker). After annealing with 5' thiol labeled complementary DNA

strands (SH-random sequence) in TM buffer, the product was added on gold surface to assemble.

In the experiment of control cell adhesion by ATP, 1μM thiol labeled DNA was incubated at 37 °C with or without the presence of 1 mM ATP for half an hour. Then DNA was grafted on the gold surface for an hour followed by passivation with SH-PEG5000 for 4 hours to perform cell experiment.

Characterization of fluorescence labeled DNA on gold surface by microscope.

DNA was assembled on gold surface as described above. Briefly, 3 μM of Cy3 labeled thiol-modified DNA in TM buffer was added on the surface for 4 h for immobilization. After passivated in 4 mM OEG for 4h, the surface was imaged with fluorescence microscopy (leica epi-fluorescence with EMCCD).

Cell experiments

Cells were seeded on PLL coated coverslip or DNA self-assemble monolayer in 24-well culture plates at a density of 6×10^4 cells /well for Hela, MCF-7 and 10×10^4 cells / well for PC12, HacaT and CHO. For ATP aptamer experiment, MCF-7 cells were seeded either with or without 1 mM ATP. At specific time point, cells were washed with pre-warmed PBS for three times, and fixed with paraformaldehyde/sucrose (4% (wt/vol)) in PBS at room temperature for 15 min. Then cells were washed with PBS and permeated with TritonX-100 (0.1%) in PBS for 15 min. After blocked with BSA (1%) for 30 min, the actin cytoskeleton were labeled with 1μg/mL of Phallodin-TRITC for 20 min and cell nuclei were labeled with Hoechst 33258 for 10 min at room temperature. Finally, the cells were washed extensively with PBS before observation by microscopy.

To study the process of cell adhesion on DNA surface, cells were fixed at various time points (0.5h, 1h, 2h, 4h,) after seeded.

Western blotting

MCF-7 cell was seeded on DNA-SAMs and PLL-coated substrates and cultured overnight. Then cells were harvested and cell numbers were counted. For protein extraction, equal numbers of cells were used. Whole lysate was resolved by SDS-PAGE, transferred onto PVDF membranes. GADPH and actin were immunoblotted by specific primary antibodies and secondary antibodies (Santa Cruz).

Statistical analysis

Cell numbers were determined by counting cell nuclei in 200 times magnified field of view and projected cell areas were determined based on an algorithm using Image J. Data are presented as mean ± SD.
